# The Factors Associated With Nonuse of Social Media or Video Communications to Connect With Friends and Family During the COVID-19 Pandemic in Older Adults: Web-Based Survey Study

**DOI:** 10.2196/34793

**Published:** 2022-06-06

**Authors:** Rachel D Savage, Sophia Di Nicolo, Wei Wu, Joyce Li, Andrea Lawson, Jim Grieve, Vivek Goel, Paula A Rochon

**Affiliations:** 1 Women's Age Lab Women's College Hospital Toronto, ON Canada; 2 Women's College Research Institute Women's College Hospital Toronto, ON Canada; 3 ICES Toronto, ON Canada; 4 Trinity College University of Toronto Toronto, ON Canada; 5 RTOERO Toronto, ON Canada; 6 Institute of Health Policy, Management and Evaluation Dalla Lana School of Public Health University of Toronto Toronto, ON Canada; 7 Division of Geriatric Medicine Department of Medicine University of Toronto Toronto, ON Canada

**Keywords:** digital technology, loneliness, older adults, COVID-19, elderly, lonely, mental health, factor, usage, social media, video, communication, connection, connect, family, friend, age, support

## Abstract

**Background:**

Digital technologies have enabled social connection during prolonged periods of physical distancing and travel restrictions throughout the COVID-19 pandemic. These solutions may exclude older adults, who are at higher risk for social isolation, loneliness, and severe outcomes if infected with SARS-CoV-2.

**Objective:**

This study investigated factors associated with nonuse of social media or video communications to connect with friends and family among older adults during the pandemic’s first wave.

**Methods:**

A web-based, cross-sectional survey was administered to members of a national retired educators’ organization based in Ontario, Canada, between May 6 and 19, 2020. Respondents (N=4879) were asked about their use of social networking websites or apps to communicate with friends and family, their internet connection and smartphone access, loneliness, and sociodemographic characteristics. Factors associated with nonuse were evaluated using multivariable logistic regression. A thematic analysis was performed on open-ended survey responses that described experiences with technology and virtual connection.

**Results:**

Overall, 15.4% (751/4868) of respondents did not use social networking websites or apps. After adjustment, male gender (odds ratio [OR] 1.60, 95% CI 1.33-1.92), advanced age (OR 1.88, 95% CI 1.38-2.55), living alone (OR 1.68, 95% CI 1.39-2.02), poorer health (OR 1.33, 95% CI 1.04-1.71), and lower social support (OR 1.44, 95% CI 1.20-1.71) increased the odds of nonuse. The reliability of internet connection and access to a smartphone also predicted nonuse. Many respondents viewed these technologies as beneficial, especially for maintaining pre–COVID-19 social contacts and routines, despite preferences for in-person connection.

**Conclusions:**

Several factors including advanced age, living alone, and low social support increased the odds of nonuse of social media in older adults to communicate with friends and family during COVID-19’s first wave. Our findings identified socially vulnerable subgroups who may benefit from intervention (eg, improved access, digital literacy, and telephone outreach) to improve social connection.

## Introduction

Digital technologies have brought people together while maintaining physical distance during the novel COVID-19 pandemic [1], with more older adults reporting use of technology to connect virtually with family and friends than ever before. The 2020 Canadian Internet Use Survey found that almost one-third (29%) of older adults aged ≥65 years reported using these services more often since the pandemic, whereas 18% indicated that the pandemic was the first time they communicated with family or friends using video platforms [2]. Although these technologies remain a lifeline to many, there are concerns that technology-based solutions may exclude many older adults, who are at higher risk for social isolation and loneliness and less likely to have the knowledge or capacity to use these technologies [3,4]. Moreover, vaccine rollouts in many regions of North America have relied on complicated websites to book appointments, require internet access and email addresses or mobile phone numbers capable of receiving text messages for registration, and use social media platforms to advertise immunization clinics. This all leads to important access barriers for older adults who are also at greater risk of severe outcomes if infected with SARS-CoV-2 [5].

Prior to the pandemic, research has shown that older adults of advanced age, men, those with lower household income and education, those living alone, those who are Black or Hispanic, or those with a disability are less likely to use the internet and health information technology [6-9]. Digital access (eg, internet access and device affordability), capacity (eg, vision or hearing issues), and an understanding of how to use these technologies are also important [3]. With the dramatically shifting landscape, what is less clear is who is being left behind in this upswell of new users. Since technology has played a vital role during this pandemic and will continue to do so postpandemic, timely data on nonusers are important to appropriately target interventions and supports. We conducted a survey among community-dwelling older Canadians to assess their use of social media or video communications to connect with friends and family and examined factors associated with their nonuse of this technology early in the COVID-19 pandemic.

## Methods

### Web-Based Survey

We conducted a closed, web-based, and cross-sectional survey with members of a national retired educators’ organization (RTOERO) between May 6 and 19, 2020. More than three-quarters of the members (76.5%, 62,000/81,000) had registered an email address with the organization and were eligible to participate. Members were invited by email from RTOERO’s chief executive officer and sent reminders at 7 and 10 days. Study materials were provided in English and French. The questionnaire was co-designed and pretested with RTOERO leadership and included 32 questions that examined the impact of COVID-19 on daily life, loneliness, and the use of digital technologies for social connectivity [10].

Social media and video communication use was measured by asking “Do you use any social networking websites (eg, Facebook) or apps (eg, Zoom or FaceTime) to communicate with friends and family?” (yes/no). Respondents were also asked about their internet connection, smartphone access, loneliness, and sociodemographic characteristics including age, gender, ethnicity, language, living arrangement, self-perceived health status and location of residence (rural/urban). The questionnaire was pretested in English with 18 RTOERO board members and staff and French with 1 staff member for usability, technical functionality, clarity, flow, sensitive questions, and timing. The pretest results were not included in the final analysis. In the pretest, it took respondents on average 13 minutes to complete the survey (median: 14 minutes).

Surveys were only analyzed if the respondent clicked “Submit” and responded to more than 1 question. Nonusers of social media were compared to users through Pearson chi-square tests in univariable analyses on sociodemographic factors (age, gender, rurality, health status, and ethnicity), access factors (internet connection and smartphone access), and relational factors (living arrangement, loneliness, communication frequency, and social support). Corresponding adjusted associations were made using multivariable logistic regression including all covariates in the model. Survey questions on gender and ethnicity included “Prefer to self-identify” or “Prefer not to say” response options, which were collapsed into an “Other” category and retained in the regression analysis. Otherwise, respondents with missing (ie, don’t know or blank) covariate values were excluded from the model.

We conducted an inductive thematic analysis of free-text responses to 4 survey questions that included an open-ended response option; these questions asked respondents to describe feelings of loneliness, strategies they use to avoid feeling lonely, how RTOERO could support members, and if they had any other comments or suggestions. Responses were reviewed for descriptions of experiences with virtual connection and a set of 14 initial codes were generated by the analyst (SDN) and discussed with the study team. Themes were identified by examining patterns across the codes and were presented, along with illustrative quotations, to both the study team and RTOERO members for input and reflection.

### Ethics Approval

Participation was voluntary, and informed consent was obtained electronically. The study was approved by the Research Ethics Board at Women’s College Hospital (#2020-0051-E) and reporting followed the Checklist for Reporting Results of Internet E-Surveys [11].

## Results

The survey completion rate among those who consented to participate was 88.8% (4891/5509). There were 12 respondents excluded who answered ≤1 question, leaving 4879 respondents in the final analysis. Most respondents were women (71%, 3421/4818), aged 65-79 years (67.4%, 3279/4863), White (91.6%, 4454/4861), and in good self-reported health (89.7%, 4370/4873; Table 1); this age and sex distribution mirrored the broader RTOERO membership (67% women and 14.5% aged <65 years, 64% aged 65-79 years, and 21.5% aged ≥80 years; personal communication by JG). Overall, 15.4% (751/4868) of respondents did not use social networking websites or apps to communicate with friends and family. Nonuse was higher in men (21.6%, 301/1394) than women (12.7%, 434/3418; *P*<.001).

A higher proportion of nonusers were men, aged ≥80 years, who lived alone and reported fair or poor health (Table 2 and Multimedia Appendix 1 by gender). These sociodemographic factors remained independently associated with nonuse of social media after adjustment. A moderate or poor internet connection and lack of smartphone access also increased the odds of nonuse, as did lower levels of social support.

Within the open-ended response data, we identified 3 relevant themes: (1) the benefits of technology, (2) maintaining pre–COVID-19 social contacts and routines, and (3) virtual connection not being a substitute for in-person connection (Table 3). References to the use of technology during the pandemic were overwhelmingly positive. Many commented that technology kept them socially connected during lockdown: “*I find I'm doing more emails and video-chats, especially with friends and relatives who live alone. Those communications benefit them and me, I feel*” (woman, age group 55-59 years). Video chat apps like Zoom and WhatsApp were valued by those living alone to alleviate loneliness: “*I am widowed so do have feelings of loneliness at times. If anything, I have had more contact on social media with others now*” (man, age group 75-79 years).

**Table 1:** Characteristics of older women and men who were survey respondents, May 2020.

Characteristic			All respondents (N=4879), n (%)		Women^a^ (n=3421), n (%)		Men^a^ (n=1397), n (%)
**Age group (years; all respondents: n=4863; women: n=3416: men: n=1395)**							
	<65	1027 (21.1)		846 (24.8)		174 (12.5)	
	65-79	3279 (67.4)		2295 (67.2)		945 (67.7)	
	≥80	557 (11.5)		275 (8.1)		276 (19.8)	
**Living arrangement (all respondents: n=4762; women: n=3356; men: n=1351)**							
	Lives alone	1415 (29.7)		1138 (33.9)		266 (19.7)	
	Does not live alone	3347 (70.3)		2218 (66.1)		1085 (80.3)	
**Location of residence (all respondents: n=4752; women: n=3348; men: n=1354)**							
	Urban	3962 (83.4)		2791 (83.4)		1132 (83.6)	
	Rural	751 (15.8)		531 (15.9)		209 (15.4)	
	Outside Canada	39 (0.8)		26 (0.8)		13 (1)	
**Self-reported health status (all respondents: n=4873; women: n=3417; men: n=1397)**							
	Excellent or very good or good	4370 (89.7)		3082 (90.2)		1238 (88.6)	
	Fair or poor	492 (10.1)		330 (9.7)		154 (11)	
	Don’t Know	11 (0.2)		5 (0.2)		5 (0.4)	
**Ethnicity (all respondents: n=4861; women: n=3410; men: n=1397)**							
	White	4454 (91.6)		3153 (92.5)		1264 (90.5)	
	Non-White	269 (5.5)		189 (5.5)		76 (5.4)	
	Other^b^	138 (2.8)		68 (2)		57 (4.1)	
**Social media use (all respondents: n=4868; women: n=3418; men: n=1394)**							
	Yes	4113 (84.5)		2983 (87.3)		1090 (78.2)	
	No	751 (15.4)		434 (12.7)		301 (21.6)	
	Don’t Know	4 (0.1)		1 (0)		3 (0.2)	

^a^61 respondents did not identify their gender.

^b^Includes respondents who selected either “Prefer to self-identify” or “Prefer not to say.”

**Table 2:** Odds ratios for nonuse of social media or video communications in a sample of older Canadians, May 2020 (N=4526).

Characteristic			Social media use			Odds ratio	
		Nonuser, n (%)		User, n (%)	Crude OR^a^ (95% CI)		Adjusted OR (95% CI)
**Gender (nonuser: n=735; user: n=4073)**							
	Men	301 (41)		1090 (26.8)	1.90 (1.61-2.23)		1.60 (1.33-1.92)
	Women	434 (59)		2983 (73.2)	ref^b^		ref
**Age group (years; nonuser: n=749; user: n=4102)**							
	<65	103 (13.8)		924 (22.5)	ref		ref
	65-79	488 (65.2)		2782 (67.8)	1.57 (1.26-1.97)		1.24 (0.98-1.57)
	≥80	158 (21.1)		396 (9.7)	3.58 (2.72-4.71)		1.88 (1.38-2.55)
**Living alone (nonuser: n=729; user: n=4020)**							
	Yes	286 (39.2)		1124 (28)	1.55 (0.96-2.49)		1.68 (1.39-2.02)
	No	443 (60.8)		2896 (72)	ref		ref
**Rural residence (nonuser: n=723; user: n=3976)**							
	Yes	105 (14.5)		646 (16.2)	0.88 (0.70-1.10)		0.90 (0.71-1.14)
	No	618 (85.5)		3330 (83.8)	ref		ref
**Self-reported fair or poor health (nonuser: n=748; user: n=4100)**							
	Excellent or very good or good	633 (84.6)		3725 (90.9)	ref		ref
	Fair or poor	115 (15.4)		375 (9.1)	1.81 (1.44-2.26)		1.33 (1.04-1.71)
**Ethnicity (nonuser: n=730; user: n=3981)**							
	White	41 (5.6)		227 (5.7)	ref		ref
	Non-White	689 (94.4)		3754 (94.3)	0.98 (0.70-1.39)		0.85 (0.59-1.22)
**Internet connection (nonuser: n=737; user: n=4078)**							
	Very good or good	647 (87.8)		3709 (91)	ref		ref
	Moderate or poor	90 (12.2)		369 (9)	1.40 (1.09-1.79)		1.39 (1.06-1.82)
**Access to a smartphone (nonuser: n=745; user: n=4098)**							
	Yes	436 (58.5)		3460 (84.4)	ref		ref
	No	309 (41.5)		638 (15.6)	3.84 (3.25-4.55)		3.08 (2.58-3.69)
**Loneliness (nonuser: n=727; user: n=4022)**							
	Always or often	82 (11.3)		321 (8)	1.37 (1.05-1.78)		1.05 (0.78-1.41)
	Some of the time	226 (31.1)		1455 (36.2)	0.83 (0.70-0.99)		0.81 (0.67-0.98)
	No	419 (57.6)		2246 (55.8)	ref		ref
**Communication frequency** **(nonuser: n=749; user: n=4102)**							
	High (≥3 times in past week)	645 (86.1)		3866 (94.2)	ref		ref
	Low (0-2 times in past week)	104 (13.9)		236 (5.8)	2.64 (2.07-3.38)		2.01 (1.54-2.62)
**Received offers of assistance** **(nonuser: n=742; user: n=4092)**							
	Yes	248 (33.4)		1660 (40.6)	ref		ref
	No	494 (66.6)		2432 (59.4)	1.36 (1.15-1.60)		1.44 (1.20-1.71)

^b^OR: odds ratio.

^a^Ref: reference category.

**Table 3:** Themes and illustrative quotes based on free-text responses to the web-based survey, May 2020.

Theme	Description	Illustrative quotes
Technology and virtual connection is beneficial for some older adults to stay connected	Considering the COVID-19 pandemic, some older adults have found using technology to stay connected virtually to be beneficial as they are unable to see people in person. They may be using technology for various activities, including, but not limited to, video calling, emailing, and messaging their friends and family.	“Zoom, What’s App etc have been excellent platforms for keeping families connected across the globe and for maintaining local social activities such as card games, yoga, book clubs etc.” “I find I'm doing more emails and video-chats, especially with friends and relatives who live alone. Those communications benefit them and me, I feel.” “Zoom app has been very helpful, able to see my mom, family members and girlfriends.”
Technology and virtual connection has allowed for some older adults to maintain connections and help to enable their routine	The internet has allowed for older adults to shift their regular activities to the web to facilitate social interactions. Some of those who were previously engaged in various activities with others were able to continue this engagement virtually during the pandemic. In this sense, the move to web-based activities is assisting in maintaining existing relationships and social connections. The theme of routine and regularity was also discussed as older adults indicated activities occurring a certain number of times a week or having scheduled calls with friends and family.	“My book club, my walking group, my outdoor club and my monthly lunch friends now meet on Zoom.” “Virtual Storytelling on-line events 3 times per week; Church groups three time per week; Book club once a week; weekly family gatherings online; daily work with political social justice groups; long walks in nature; great friends; lots of exercise scheduled at the same time daily” “I participate in a virtual exercise class twice a week, virtual bridge club twice a week and Facetime chats twice a week. Family zoom calls periodically. I text a friend that I am coming by and walk past her house and wave and talk through an open window.”
Technology and virtual connection is not a replacement for social interaction	Although technology and virtual connection has been positive for some, there is still critiques that it is not the same as or a replacement for in-person interactions. Respondents indicated that although they are using virtual connection or technology to stay connected now, they still long for that in-person interaction and connection.	“Although I've connected with family and friends by telephone, it isn't the same as face to face” “Virtual book club however human closeness and touching (i.e. hugs) is imperative for high quality living” “I try to reach out via social media to my best friend daily. I phone family members who are not on social media. However, regardless of the advances of social media, nothing will ever replace face-to-face contact or the human touch.”

Women described how technology allowed them to shift their regular activities to the web to maintain pre—COVID-19 routines: “*My book club, my walking group, my outdoor club and my monthly lunch friends now meet on Zoom*” (woman, age group 70-74 years). For others, social networking technology was used to establish new social routines.

Despite this, some respondents acknowledged the limitations of these technologies, still longing for in-person interaction: “*texting and phone calls are great, but the one-on-one contact is missing*” (man, age group 75-79 years) and “*regardless of the advances of social media, nothing will ever replace face-to-face contact or the human touch*” (woman, age group 65-69 years).

## Discussion

### Principal Findings and Comparison With Prior Work

In a survey of 4879 older retirees of the education sector, we found that 1 in 7 were not using social networking websites or apps to communicate with friends and family early in the COVID-19 pandemic. We identified several characteristics associated with an increased likelihood of nonuse including male gender, advanced age, living alone, poorer health, poor internet connection, a lack of smartphone access, and lower social support. Our findings are consistent with studies of technology use in older adults, which similarly show that advanced age, male gender, being unmarried, and poorer self-reported health are important predictors of nonuse and telemedicine unreadiness [3,12].

We found that sentiments toward technology use during the early weeks of the pandemic were mostly positive, with respondents describing how technology facilitated social connections, allowed them to maintain routines, or helped them adjust to a new way of life. Qualitative studies on the experiences of older adults during the pandemic’s first wave report similar findings, with technology being described as having a facilitative role in allowing older adults to engage in the things that matter most to them despite ongoing restrictions [13-15].

### Implications

Loneliness and social isolation are top concerns of older adults during the COVID-19 pandemic [10,16], so developing effective strategies to maintain social connection while adhering to public health recommendations is essential. For many older adults, digital communication has been a source of joy and comfort during the often uncertain times of the pandemic [16]. Efforts are needed to enable access and build digital literacy to support those who wish to use these technologies but are currently unable to. This includes rigorous research on what strategies are effective and for whom and addressing structural barriers to technology use, including universal internet access [4]. Such improvements could have far-reaching impacts beyond enhanced social connection. Now more than ever, digital technologies are being used to deliver lifesaving health care and health promotion services, including access to COVID-19 testing and vaccines and other essential services including education, groceries, banking, shopping, and government resources and support. Improving the adoption and use of these technologies in older adults and removing structural barriers to access will ensure the benefits of technology are equitably realized. This is especially important as telehealth expands and is incorporated into routine clinical practice [12].

Despite its benefits, technology-based solutions are not a panacea for social connection. In our study and others [17], older adults have acknowledged that even with virtual connection, gaps in social connection and support remain. A recent comparative study using nationally representative longitudinal survey data in the United States and United Kingdom found that virtual contact during the pandemic did not compensate for the lack of in-person contact in terms of supporting older adults’ mental well-being in either country [18]. In fact, the authors found that in the United States, older adults with more frequent virtual contact were more likely to feel lonely during the pandemic and have become lonelier in both countries [18]. Although we did not find an association between loneliness and social media use/nonuse, future research is needed to better understand temporal associations and the potential unintended consequences of virtual connection, particularly for subgroups like older women, who have been reported to be more likely to rely on virtual-only contact during the pandemic [18].

These findings underscore that the value of in-person and offline connections cannot be forgotten. Weekly telephone call interventions with volunteers have shown benefit to both older adults living in long-term care [19] and those in the community [20] during the COVID-19 pandemic. Supporting older adults to remain socially connected through more traditional means is an important and more accessible complement to technological solutions; both are needed to ensure solutions to the issue of loneliness do not further exclude older adults who are not on the internt [4,21,22]. Outdoor environments and spaces are a safe option for connecting in-person at a distance, and the use of these spaces have been shown to be an important predictor of social connectedness pre–COVID-19 [23]. Improving equitable access to safe, accessible open spaces within neighborhoods and enhancing existing ones to better facilitate social interaction would again pay dividends now and in the future.

### Limitations

Our study has several limitations. Our study is based on a convenience sample of community-dwelling, retired educators who had internet access. Due to this sample, the prevalence of social media use was likely overestimated in our study. Estimates of social media use by older adults during the pandemic for social connection are wide-ranging (eg, 37% to 80% based on surveys using similar methodologies conducted during the first wave in the United States and Canada) [24,25]. The rates of use are expected to be lower in the general population of older adults, who may face access barriers due to lower levels of education, lower income, health challenges, and ethnicity or race. In a racially diverse study of US older adults, 76% reported minimal video-based socializing; access to and discomfort with technology were described as a key barrier to coping, maintaining social connection, and accessing health care during the pandemic [26]. There are other factors that are associated with nonuse of digital technologies that we did not evaluate, including cognitive impairment, frailty, vision or hearing impairments, and non-English/French speaking ability [27,28]. The unique needs and barriers of these population subgroups warrant thorough investigation and consideration when designing interventions, programs, and services.

### Conclusion

Among a sample of retired educators with internet access, we found that male gender, advanced age, living alone, poorer health, and lower social support increased the odds of nonuse of social media to communicate with friends and family during the first wave of the COVID-19 pandemic. The reliability of internet connection and smartphone access also predicted nonuse. Strategies to improve the uptake of digital technologies for social connection must address structural barriers to accessing technology and build digital literacy among older adults. Complementary approaches, such as telephone outreach, are also needed to improve social connection without further excluding older adults who remain offline.

## Appendix

Multimedia Appendix 1 Characteristics of social media users and nonusers, stratified by gender, May 2020.

## Abbreviations

OR: odds ratio
