# Supplementary material for: The Factors Associated With Nonuse of Social Media or Video Communications to Connect With Friends and Family During the COVID-19 Pandemic in Older Adults: Web-Based Survey Study
Source: JMIR Aging. 2022 Jun 6;5(2):e34793. doi: 10.2196/34793 (PMC9177172; doi:10.2196/34793)
Supplement: Multimedia Appendix 1 [file aging_v5i2e34793_app1.docx]

**Supplementary Table S1**. Characteristics of social media users and non-users, stratified by gender, May 2020.

|  | Women | |  | Men | |  |
| --- | --- | --- | --- | --- | --- | --- |
|  | Social media/app use | |  | Social media/app use | |  |
| Characteristic | Yes | No | *P* value | Yes | No | *P* value |
| **Age Group, years** |  |  |  |  |  |  |
| <65 | 776 (26.1%) | 70 (16.1%) | <.001 | 142 (13.1%) | 32 (10.6%) | .002 |
| 65-79 | 2004 (67.3%) | 288 (66.4%) |  | 753 (69.2%) | 188 (62.5%) |  |
| 80+ | 198 (6.6%) | 76 (17.5%) |  | 193 (17.7%) | 81 (26.9%) |  |
| **Living alone** |  |  |  |  |  |  |
| Yes | 925 (31.6%) | 210 (49.4%) | <.001 | 191 (18.1%) | 74 (25.6%) | .004 |
| No | 2002 (68.4%) | 215 (50.6%) |  | 866 (81.9%) | 215 (74.4%) |  |
| **Rural residence** |  |  |  |  |  |  |
| Yes | 470 (16.2%) | 61 (14.4%) | .331 | 169 (16.1%) | 40 (14.0%) | .381 |
| No | 2424 (83.8%) | 363 (85.6%) |  | 880 (83.9%) | 246 (86.0%) |  |
| **Self-reported health** |  |  |  |  |  |  |
| Excellent, very good or good | 2713 (91.2%) | 367 (84.8%) | <.001 | 979 (90.2%) | 253 (84.3%) | .005 |
| Fair or poor | 262 (8.8%) | 66 (15.2%) |  | 107 (9.8%) | 47 (15.7%) |  |
| **Ethnicity** |  |  |  |  |  |  |
| Non-white | 162 (5.6%) | 27 (6.4%) | .510 | 61 (5.9%) | 14 (4.8%) | .487 |
| White | 2751 (94.4%) | 398 (93.6%) |  | 981 (94.1%) | 278 (95.2%) |  |
| **Internet connection** |  |  |  |  |  |  |
| Very good or good | 2696 (91.2%) | 365 (86.3%) | .001 | 981 (90.5%) | 267 (89.6%) | .642 |
| Moderate or poor | 260 (8.8%) | 58 (13.7%) |  | 103 (9.5%) | 31 (10.4%) |  |
| **Access to a smartphone** |  |  |  |  |  |  |
| Yes | 2564 (86.2%) | 261 (60.8%) | <.001 | 866 (79.7%) | 168 (56.0%) | <.001 |
| No | 409 (13.8%) | 168 (39.2%) |  | 220 (20.3%) | 132 (44.0%) |  |
| **Loneliness** |  |  |  |  |  |  |
| Always or often | 259 (8.8%) | 55 (12.9%) | .019 | 57 (5.4%) | 26 (9.0%) | .081 |
| Some of the time | 1199 (40.9%) | 158 (37.2%) |  | 242 (23.0%) | 65 (22.5%) |  |
| No | 1472 (50.2%) | 212 (49.9%) |  | 754 (71.6%) | 198 (68.5%) |  |
| **Communication frequency** |  |  |  |  |  |  |
| High (≥3 times in past week) | 2840 (95.5%) | 389 (89.8%) | <.001 | 990 (91.1%) | 242 (80.4%) | <.001 |
| Low (0-2 times in past week) | 135 (4.5%) | 44 (10.2%) |  | 97 (8.9%) | 59 (19.6%) |  |
| **Received offers of assistance** |  |  |  |  |  |  |
| Yes | 1258 (42.4%) | 169 (39.4%) | .236 | 391 (36.0%) | 79 (26.5%) | .002 |
| No | 1708 (57.6%) | 260 (60.6%) |  | 696 (64.0%) | 219 (73.5%) |  |
